# DrugTax: package for drug taxonomy identification and explainable feature extraction

**DOI:** 10.1186/s13321-022-00649-w

**Published:** 2022-10-27

**Authors:** A. J. Preto, Paulo C. Correia, Irina S. Moreira

**Affiliations:** 1 Center for Neuroscience and Cell Biology, University of Coimbra, 3004-504 Coimbra, Portugal; 2 PhD Programme in Experimental Biology and Biomedicine, Institute for Interdisciplinary Research (IIIUC), University of Coimbra, Casa Costa Alemão, 3030-789 Coimbra, Portugal; 3 Department of Life Sciences, University of Coimbra, Calçada Martim de Freitas, 3000-456 Coimbra, Portugal; 4 CIBB - Center for Innovative Biomedicine and Biotechnology, University of Coimbra, 3004-504 Coimbra, Portugal

**Keywords:** DrugTax, Small molecules, Machine learning, Explainability, Python

## Abstract

DrugTax is an easy-to-use Python package for small molecule detailed characterization. It extends a previously explored chemical taxonomy making it ready-to-use in any Artificial Intelligence approach. DrugTax leverages small molecule representations as input in one of their most accessible and simple forms (SMILES) and allows the simultaneously extraction of taxonomy information and key features for big data algorithm deployment. In addition, it delivers a set of tools for bulk analysis and visualization that can also be used for chemical space representation and molecule similarity assessment. DrugTax is a valuable tool for chemoinformatic processing and can be easily integrated in drug discovery pipelines. DrugTax can be effortlessly installed via PyPI (https://pypi.org/project/DrugTax/) or GitHub (https://github.com/MoreiraLAB/DrugTax).

**Graphical Abstract:**

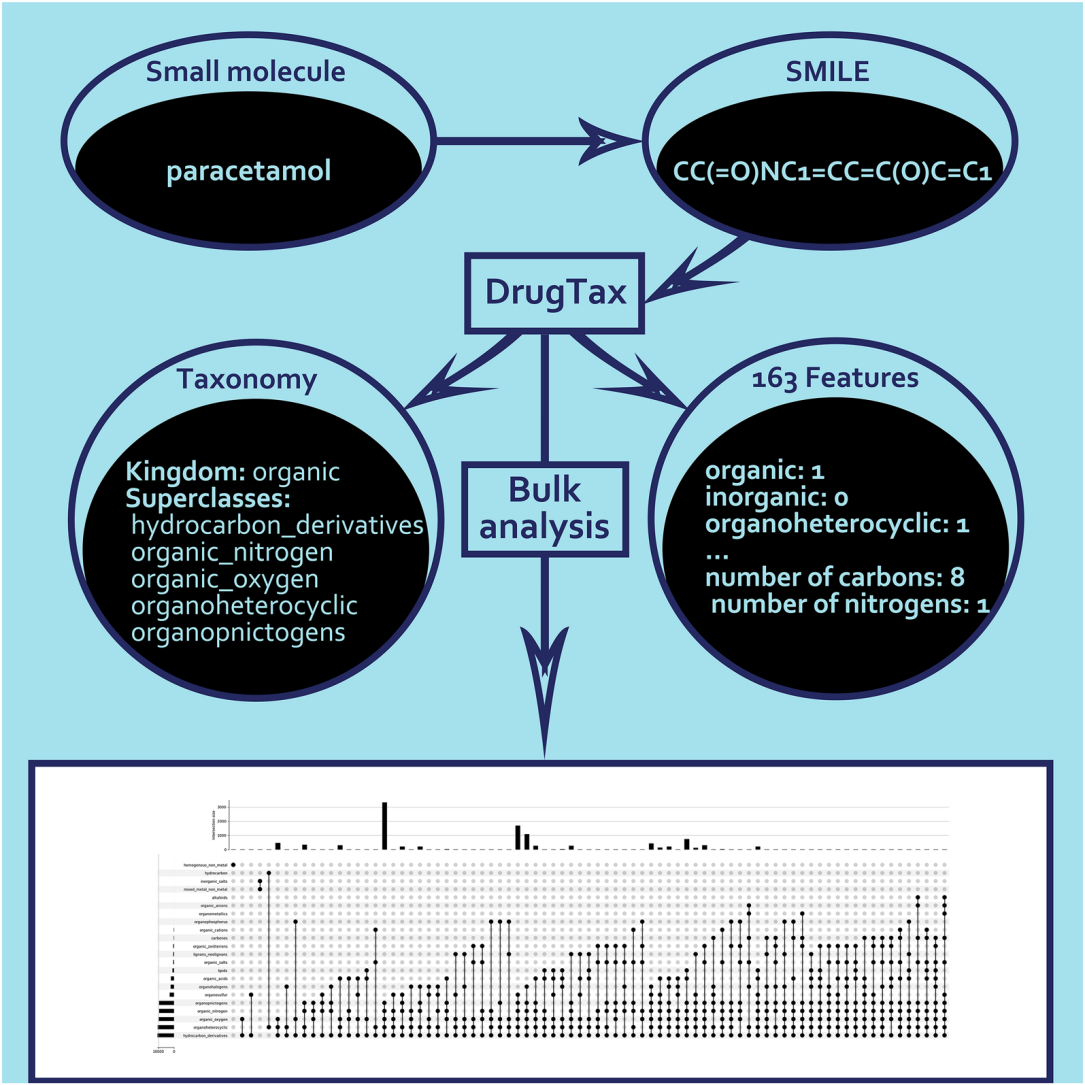

**Supplementary Information:**

The online version contains supplementary material available at 10.1186/s13321-022-00649-w.

## Introduction

PubChem [1] registers over 111 million compounds and 278 million substances (August 2022). According to Drugbank [2] there are 2725 approved drugs, among 11,937 possible drugs. ChEMBL [3] reports over 2.2 million compounds and 14,000 drugs. The abundance of drugs or drug-like compounds is evidently overwhelming, which is often problematic, when considering automatized approaches.

The surge of Artificial Intelligence (AI) and its subfield Machine Learning (ML) to tackle problems involving drugs or, overall, small ligands has been significant in the last few years [4]. For this purpose, it is advantageous to be able to provide a deeper understanding of the drugs’ characteristics while also being able to numerically describe them [5]. Feature extraction is a focus when considering ML-based approaches, as it is a crucial and necessary step for any algorithms to be able to distinguish between the different patterns within the data. Under the scope of drug discovery, several packages have been developed to this end. Open Babel [6] is a broad example, providing a set of chemical tools to describe and manipulate drugs and other small molecules. More recently, packages such as Mordred [7] or ChemmineR [8] have also been developed. Alternatively, a different type of approaches can also be used for ML processing, such as the ones based on graph [9, 10] and voxel-based [11] drug representations. The chemical characterization of small molecules is a cornerstone for further understanding and essential for bulk data approaches, and as such we explored the usage of this type of knowledge for data grouping and feature extraction, some of the characterizations stemming from the root biochemical definitions [12].

Our new developed Python package, DrugTax, follows the definitions made available by ChemOnt and Classyfire [13]. The Classyfire protocol [13] is very useful for small molecule taxonomy classification as it performs a levelled classification in 11 different levels (Kingdom, SuperClass, Class, SubClass, etc.), yielding over 4800 different categories. We also explored the chemical ontology (ChemOnt), developed by the same authors, which allows the classification of the small molecules solely by rule-based steps. However, these protocols still presented some shortcomings: (i) the API, although properly documented, is faulty in bulk submissions; (ii) although both the browser and the API are available, the ChemOnt code for small molecule taxonomic classification is not accessible, limiting the users to using the authors’ API; and finally, (iii) while the same-level categories are not necessarily mutually exclusive, Classyfire [13] yields a single classification for each compound. This means that molecules belonging to more than one superclass, are overlooked, leading to major oversights of information when considering multiple molecules’ comparison. These shortcomings are particularly relevant if the research’s main aim is to group small ligands according to their characteristics.

DrugTax solves that problem by allowing the user to install and inspect the code that generates the small molecules classes in an easy-to-use package. DrugTax provides the prior classification between the two possible kingdoms, organic and inorganic, and, respectively, their 26 and 5 superclasses. These superclasses are returned in the form of a list, thus allowing overlapping superclasses. Subsequently, DrugTax displays UpSet plots [14], which are ideal for identifying and inspecting large volumes of intersecting sets to provide the user an approach to further tailor the groupings to their needs. Finally, DrugTax provides an option to use features derived from the taxonomic analysis up until superclasses. This innovation can be promptly used for ML purposes or simply small molecule data visualization.

## Methods and implementation

**Fig. 1:**
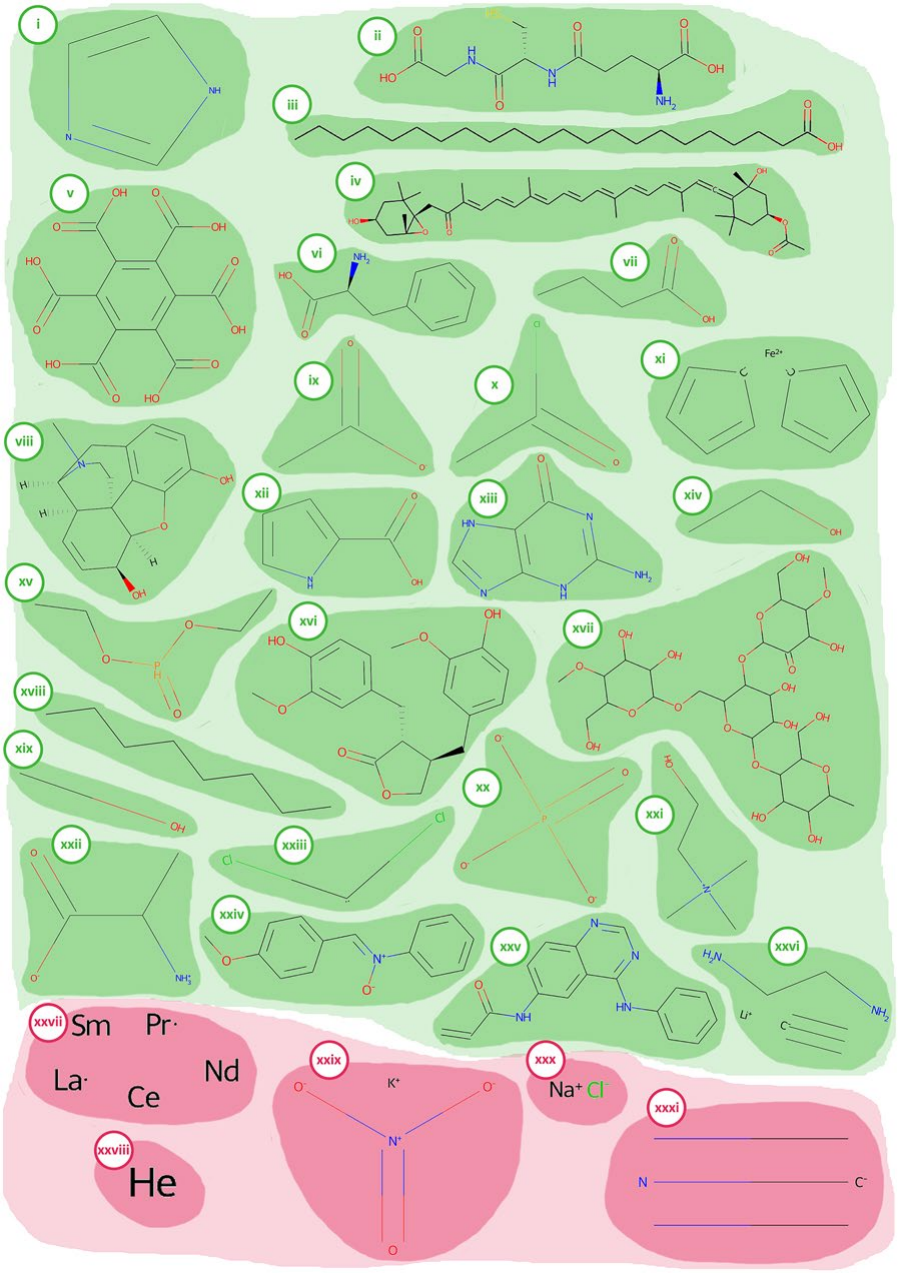
Graphical representation of each of the 31 superclasses. Organic molecules are highlighted in green, while inorganic molecules are shown in red. The molecules depicted are: organoheterocyclic-imidazole (**i**); organosulphur-glutathione (**ii**); lipid molecule-behenic acid (fatty acid) (**iii**); allene-fucoxanthin (**iv**); benzenoid-benzene hexacarboxylic acid (**v**); phenylpropanoid-phenylalanine (**vi**); organic acid-butyric acid (**vii**); alkaloid-morphine (**viii**); organic salt-acetate (**ix**); organohalogen-acetyl chloride (**x**); organometallic-ferrocene (**xi**); organic nitrogen-pyrrole-2-carboxylate (**xii**); nucleotide-guanine (**xiii**); organic oxygen-ethanol (**xiv**); organophosphorus-diethyl phosphonate (**xv**); lignans and neolignans-matairesinol (**xvi**); organic polymer-starch (**xvii**); hydrocarbon-octane (**xviii**); hydrocarbon derivative-ethanol (**xix**); organic anion-phosphate (**xx**); organic cation-choline (**xxi**); organic zwitterion-ammonium propionate (**xxii**); carbene-dichlorocarbene (**xxiii**); organic 1,3-dipolar-nitrone molecule (**xxiv**); organopnictogen-N-(4-phenylamino-quinazolin-6-yl)-acrylamide (**xxv**); acetylide-lithium acetylide (**xxvi**); homogenous metal - cerium with mixed metals (**xxvii**); homogenous non-metal-noble gas helium (**xxviii**); mixed metal/non-metal-potassium nitrate (**xxix**); inorganic salt-sodium chloride (**xxx**); miscellaneous inorganic-cyanide (**xxxi**)

DrugTax is centered around a Python object class that takes as input a Simplified Molecular Input Line Entry System (SMILES) [15] and computes several necessary steps for the upcoming kingdom and superclass assignment. If a SMILES representation is not provided, DrugTax will default to download its isomeric form from a provided name. All Code Snippets (C.S.) can be found in Additional file 1. Figure 1 illustrates molecules belonging to the 31 superclasses that will be listed next. Organic molecules are highlighted in green, while inorganic molecules are shown in red.

### DrugTax class, helper functions and variables

Prior to starting the calculations, a few variables (C.S.1—Halogens, metals and group-15/nitrogen atoms lists) helper functions were constructed (C.S.2—To retrieve only ordered atom sequence and C.S.3—To allow atom rings identification). Furthermore, two functions were made available for upcoming feature extraction: one allows for the count of characters on SMILES (C.S.4), while the other initializes an empty dictionary of superclass feature data (C.S.5). Finally, the DrugTax class object itself is initialized with the computation of several useful characteristics (C.S.6 – DrugTax class object initialization).

### Kingdoms: organic and inorganic

The general rule to assess whether a compound is organic, or inorganic depends on the existence of at least one carbon atom, in which case it is categorized as an organic compound. There are a few exceptions. For example, some compounds, although containing carbon atoms, are nonetheless, considered inorganic, e.g., isocyanide/cyanide, thiophosgene, carbon diselenide, carbon monosulphide, carbon disulphide, carbon subsulphide, carbon monoxide, carbon suboxide and dicarbon monoxide. The code accessible in C.S.7 allows the discrimination between the two possible kingdoms. Subsequently the matching superclasses will be called, in accordance with C.S.6.

### Organic compounds

As previously mentioned, an in accordance with ClassyFire [13], DrugTax considers 26 possible superclasses for organic compounds, listed below and for which the code to compute them from the basic SMILES is displayed in Additional file 1.

### Organoheterocyclic

According to the Nomenclature of Organic Compounds “*Organic heterocyclic systems contain one or more foreign elements such as oxygen, sulphur, or nitrogen in addition to carbon*” [16]. As such, we considered organoheterocyclic compounds those which contain a ring with least one carbon atom and one non-carbon atom (C.S.8). The organoheterocyclic superclass is illustrated with an imidazole molecule in Fig. 1-i.

#### Organosulphur

According to Arya et al*.* [17], “*Organosulphur compounds are organic molecules that contain sulphur and are associated with the pungent odors*” [17], and as such, we identified organosulfur compounds as those with at least one carbon–sulphur bond (C.S.9). The organosulphur superclass is depicted with a glutathione in Fig. 1-ii.

#### Lipids

According to the definition by Jones [18], “*Lipids may be classified as a mixed group of substances with the common characteristics of solubility in organic solvents*”. This group of biological molecules can be further split into simple lipids (i), such as fats—neutral esters of glycerol with satured and unsaturated acids; compound lipids (ii) consist of a fatty acid, an alcohol and at least one group containing atoms such as phosphorus or nitrogen; derived lipids (iii) are fatty acids that stem from simple or compound lipids by means of hydrolysis.

As seen above, the chemical definition of lipids is quite broad. Within DrugTax implementation, we narrowed it down to fatty acids and their derivatives, as well as substances related biosynthetically or functionally to these compounds. This corresponds to the occurrence of carboxyl group as well as a carbon chain at least four carbons long, regardless of chain saturation (C.S.10). These criteria were driven by literature assessment, in agreement with Aslan and Aslan, 2017 definition [19]. Behenic acid (fatty acid) is shown in Fig. 1-iii.

#### Allenes

“*Allenes are organic compounds in which one carbon atom has double bonds with each of its two adjacent carbon centres*” in accordance with IUPAC Gold Book allenes entry [20]. The definition includes both the hydrocarbon molecules and their derivatives obtained by substitution (C.S.11). The allenes superclass is depicted with a fucoxanthin in Fig. 1-iv.

#### Benzenoids

According to Gutman and Babić [21], benzenoids are aromatic compounds containing one or more benzene rings, formed solely by carbon atoms. The code for benzenoid superclass attribution can be consulted at C.S.8. Benzene hexacarboxylic acid, an example, is representated in Fig. 1-v.

#### Phenylpropanoids and polyketides

According to Zhang and Stephanopoulos [22], “The phenylpropanoids are a family of organic compounds with an aromatic ring and a three-carbon propene tail and are synthesized by plants from the amino acids phenylalanine and tyrosine” [23]. Regarding polyketides, Korman et al. says: “Polyketides are a large class of structurally diverse, acetate derived natural products that exhibit a wide range of bioactivities.” [24]. As such, phenylpropanoids and polyketides are organic compounds that are synthesized either from the amino acid phenylalanine (phenylpropanoids) or the decarboxylative condensation of malonyl-CoA (polyketides). Phenylpropanoids are aromatic compounds based on the phenylpropane skeleton. Polyketides usually consists of alternating carbonyl and methylene groups (beta-polyketones), biogenetically derived from repeated condensation of acetyl coenzyme A (via malonyl coenzyme A) (C.S.12). The phenylpropanoids and polyketides superclass is depicted with a phenylalanine in Fig. 1-vi.

#### Organic acids and derivatives

According to Richter et al. [25] “Organic acids are weak acids with pK_a_ values that range widely from as low as 3 (carboxylic) to as high as 9 (phenolic)”. Furthermore, according to Papagianni 2011, “Organic acids contain one or more carboxylic acid groups, which may be covalently linked in groups such as amides, esters, and peptides.” Although we are aware that there are different definitions, some of which consider organic acids without a carboxyl group [26], we considered organic acids those with carboxyl groups (C.S.13). The organic acids superclass is depicted using butyric acid as an example in Fig. 1-vii.

#### Alkaloids

According to Kurek, “Alkaloids are a huge group of naturally occurring organic compounds which contain nitrogen atom or atoms (amino or amido in some cases) in their structures. These nitrogen atoms cause alkalinity of these compounds” [27]. DrugTax classifies small molecules as alkaloid it exists nitrogen atom(s) and they have a negative net charge (C.S.14). The alkaloid superclass is depicted with a morphine molecule in Fig. 1-viii.

#### Organic salts

Organic compounds consist of an assembly of cations and anions, of which one must be organic. According to Seçken, Nilgün, “Organic salts, however, are compounds that are formed from at least one anion and one cation. Their anions are organic acid based” [28] (C.S.15). Acetate molecule was used to exemplify this superclass in Fig. 1-ix.

#### Organohalogen compounds

According to Roberts and Caserio. “*The general term of "organohalogen" refers to compounds with covalent carbon-halogen bonds*” [29]. As such, by listing the halogen atoms in C.S.1, using the code below it is possible to identify organohalogens (C.S.16). The organohalogen compounds superclass is depicted with an acetyl chloride in Fig. 1-x.

#### Organometallic compounds

According to Abbot et al*.* the existence of at least on metal–carbon allows the classification into Organometallic compounds [30]. Given this definition, DrugTax identifies organometallic compounds using the same code as for organohalogens (C.S.16) but accessing the metals list instead (C.S.1). The organometallic compounds superclass is depicted with ferrocene in Fig. 1-xi.

#### Organic nitrogen compounds

According to Moreno and Peinado, “Nitrogen compounds can be classified as mineral or organic. (…) Organic compounds, in contrast, are carbon and hydrogen compounds that contain a nitrogen atom” [31]. In the context of DrugTax, organic nitrogen compounds are simply organic compounds that contain nitrogen atoms. As such, we identify nitrogen atoms upon kingdom attribution completion (C.S. 17). Pyrrole-2-carboxylate, an example of this superclass, can be found in Fig. 1-xii.

#### Nucleosides and nucleotides

According to Sparkman et al. “*Nucleosides consist of a purine or a pyrimidine base and a ribose or a deoxyribose sugar connected*” [32]. Nucleotides, on the other hand, are defined by Joseph, A. as “*A nucleotide is a subunit of DNA or RNA that consists of a nitrogenous base (A, G, T, or C in DNA; A, G, U, or C in RNA), a phosphate molecule, and a sugar molecule (deoxyribose in DNA, and ribose in RNA)*” [33]. Considering these definitions, nucleotides are simply nucleosides with phosphate groups. As such, to identify nucleosides and nucleotides is necessary to encounter any combination of cytosine, adenine, guanine, thymine, uracil with either ribose or deoxyribose (C.S.18). The nucleosides and nucleotides superclass are represented with guanine in Fig. 1-xiii.

#### Organic oxygen compounds

As shown by Lee and Meyer [34], the quantification of oxygen in organic compounds can be detrimental in characterizing said compounds. DrugTax also identifies whether the input drug has oxygens or not (C.S.17). The organic oxygen compounds superclass is illustrated with ethanol Fig. 1-xiv.

#### Organophosphorus compounds

According to Müller “*Organophosphorus compounds with phosphorus–carbon multiple bonds provide a rich and fascinating coordination chemistry*” [35]. By identifying phosphorus in an organic compound (C.S.17), we can recognize organophosphorus compounds. The organophosphorus compounds superclass is depicted with diethyl phosphonate Fig. 1-xv.

#### Lignan and neolignans

Sang and Zhu states: “*Lignans form a group of phenolic compounds with a backbone of two phenylpropanoid (C6C3) units*” [36]. According to this definition, DrugTax identifies lignans and neolignans according to the occurrence of either p-propyphenol or phenylpropane (C.S.19). The lignans and neolignans superclass is shown with matairesinol Fig. 1-xvi.

#### Organic polymers

Yadav and Sinha states that organic polymers are long, chained macromolecules composed of many repeating monomer units” [37]. As such, DrugTax identifies repeating patterns in the molecules of the organic kingdom to identify organic polymers (C.S.20). The organic polymers superclass is depicted with starch Fig. 1-xvii.

#### Hydrocarbons

According to Enerijiofi “*Hydrocarbons are a group of chemical organic compounds composed of carbon and hydrogen*” [38]. In this case, if the input molecule has not atoms besides carbon and hydrogen, DrugTax will classify the molecule as a hydrocarbon (C.S.21). The hydrocarbons superclass is depicted with octane Fig. 1-xviii.

#### Hydrocarbon derivatives

Extending from the definition of Enerijiofi, hydrocarbon derivatives are organic compounds derived from hydrocarbon in which there are atoms different from carbon and hydrogen. DrugTax uses the same function (C.S.21) to identify both hydrocarbons and hydrocarbon derivatives. The hydrocarbon derivatives superclass is portrayed with ethanol Fig. 1-xix.

#### Organic anions

According to Sekine et al*.*:”*Organic anions are chemically heterogeneous substances possessing a carbon backbone and a net negative charge*” [39]. As such, DrugTax accounts identifies as organic cations the organic molecules with a negative net charge (C.S.22). The organic anions superclass is showed with phosphate Fig. 1-xx.

#### Organic cations

In contrast with Sekine et al*.*’s definition of organic anions, organic cations carry a net positive charge. As such, the same process can be applied (C.S.22), this time considering an overall positive net charge. The organic cations superclass is shown with choline Fig. 1-xxi.

#### Organic zwitterions

According to Hadjesfandiari and Parambath: “*Zwitterions contain both positive- and negative-charged groups, with an overall neutral charge*“ [40]. Considering this definition, DrugTax leverages the same approach of the previous two superclasses (C.S.22), for organic cations and anions. However, in this case, it is important to highlight that zwitterions are not merely organic compounds without a charge. They must have an equal number of negative and positive charges. The organic zwitterions superclass is depicted with ammonium propionate in Fig. 1-xxii.

#### Carbenes

Savin states: “*A carbene* *is a neutral divalent carbon species containing two electrons that are not shared with other atoms*” [41]. As such, DrugTax identifies carbenes as organic molecules with unpaired electrons at a carbon atom (C.S.23). The carbenes superclass is depicted by dichlorocarbene in Fig. 1-xxiii.

#### Organic 1,3-dipolar compounds

The IUPAC Compendium of Chemical Terminology defines dipolar compounds as “*Electrically neutral molecules carrying a positive and a negative charge in one of their major canonical descriptions*” [42]. Further along, it extends the definition to 1,3-dipolar compounds as “*those in which a significant canonical resonance form can be represented by a separation of charge over three atoms*” [42]. According to this definition, DrugTax identifies organic 1,3-dipolar compounds if they simultaneously possess positive and negative charges. However, the net charge should be neutral, and the compound must have one atom separating the atoms with the opposing charges (C.S.24). Nitrone molecule was chosen as an example, and it is depicted in Fig. 1-xxiv.

#### Organopnictogen compounds

IUPAC defines pnictogens as an atom belonging to group 15 of the periodic table, which include nitrogen, phosphorus, arsenic, antimony and bismuth [43]. To identify organopnictogens, DrugTax leverages the list of the group 15 atoms (C.S.1) and checks whether there are any bounds between these atoms and carbons (C.S.25). The organopnictogen superclass is depicted with N-(4-phenylamino-quinazolin-6-yl)-acrylamide in Fig. 1-xxv.

#### Acetylides

According to the IUPAC Compendium of Chemical Terminology, acetylides obey the following principles: “Compounds arising by replacement of one or both hydrogen atoms of acetylene (ethyne) by a metal or other cationic group. E.g., NaC≡CH monosodium acetylide. By extension, analogous compounds derived from terminal acetylenes, RC≡CH” [44]. By using the list of metal atoms (C.S.1), DrugTax identifies acetylides as organic compounds with a triple covalent bond between two carbon atoms, with at least one of them, bounded to a metal atom (C.S.26). Lithium acetylide is portrayed as an example of this superclass in Fig. 1-xxvi.

### Inorganic

As previously mentioned, and in accordance with ClassyFire [13], DrugTax considers five possible superclasses for inorganic compounds, listed in the next subsections. As these definitions are overall quite straightforward and elementary, we will present equally simple definitions.

#### Homogenous metal compounds

Homogenous metal compounds are inorganic compounds that contain only metal atoms. These atoms, however, are not necessarily all atoms of the same metal. The list of metals was retrieved from C.S.1. The code to identify homogenous metal compounds can be found at C.S.27. The homogenous metal superclass is illustrated as cerium with mixed metals Fig. 1-xxvii.

#### Homogenous non-metal compounds

Homogenous non-metal compounds are inorganic compounds that contain only non-metal atoms. The list of metals was retrieved from C.S.1. The code to identify homogenous non-metal compounds can be found at C.S.28. As an example, gas helium is shown in Fig. 1-xxviii.

#### Mixed metal/non-metal compounds

Mixed metal/non-metal compounds are inorganic compounds that can contain simultaneously metal and non-metal atoms. The list of metals was retrieved from C.S.1. The code to identify homogenous non-metal compounds can be found at C.S.29. Potassium nitrate is depicted as an example in Fig. 1-xxix.

#### Inorganic salts

The superclass of inorganic salts consists of inorganic compound with one or more charges, either negative or positive ones. The code to identify inorganic salts can be found at C.S.30. The inorganic salts superclass is depicted with sodium chloride in Fig. 1-xxx.

#### Miscellaneous inorganic compounds

The identification of miscellaneous inorganic compounds is dependent on the previous four inorganic superclasses. If a given compound does not fit any of these superclasses, it is considered a miscellaneous inorganic compound. Cyanide (Fig. 1-xxxi) was chosen to illustrate this superclass.

### DrugTax bulk analysis and plotting tools

One of the main purposes of this work was to allow bulk analysis of chemical properties of drugs to enable proper, tailored, and comprehensive categorization of small ligands. With that in mind, DrugTax has an additional tool for bulk ligand analysis, which makes use of kingdom and superclass attribution to perform categorization of small molecules. These categories account for multiple superclasses, in the cases in which this is possible. Firstly, it was added a short functionality to fetch the isomeric SMILES from the drug name, by using pubchempy (C.S.31). Then, using C.S. 1–30, the different superclasses for each ligand are listed (C.S.32).

By retrieving summary data from the input list of SMILES, DrugTax uses individual small ligand information to generate a fast characterization tool of small molecule datasets. Furthermore, by making use of UpSetPlot [14], DrugTax can depict many intersecting sets (in the form of small ligand superclasses), which is often limited by more conventional forms of visualization. The plots are generated from the summary information previously retrieved and can be tuned to avoid close to empty superclass aggregations (C.S.33).

## Results and case study

To exemplify the usage of DrugTax, we developed a short approach that assembles a dataset focused on drugs associated with a variety of known viruses. Firstly, we performed a query using PUG-REST (Power User Interface–Representational State Transfer) [45], a web interface of PubChem [1] that allows the programmatic access of information of chemical compounds present in the database. The requests to the server are made through URLs (Uniform Resource Locators). To comply with PUG-REST’s request volume limit, 100 compounds are fetched at a time, while the total amount of compounds to be analyzed must be specified by the user. This parameter ultimately affects the size of the resulting dataset. The compounds are scraped by the iterating over the list of CIDs (Compound ID).

Another parameter that must be specified by the user are the keywords related to the dataset one wants to create. These keywords must be present in the more relevant bioassays titles, in this case, the keywords were chosen after looking at the most frequently appearing terms in the titles of *Journal of Virology* [46] studies (accessed on the 29th of July 2022). The chosen keywords affect the size, diversity, and quality of the dataset, and so a good selection is key. It is also to note that these keywords are case sensitive and can also be present inside a word. The used keywords were: DENV, HIV, H1N1, virus, viral, Viral, SARS, Virus, HCV, influenza, Influenza, HSV, HHV, EBOV, MERS. This query was performed over 700.000 compounds.

To build a dataset relevant in the settings of both a biological problem and ML implementation, it was relevant to narrow the compounds according to their activity. As such, we selected only compounds that were featured in biological activity studies. To fulfill these criteria, we explored the information related to bioassays, regarding our compounds, in PubChem [1]. Bioassays are analytical methods to calculate the potency of chemical compounds in biological beings, making them a good source of experimentally proven data that can be accessed easily through PUG-REST [45]. We retrieved the corresponding bioassays for each compound.

Regarding the bioassays that were relevant for DrugTax’s purpose a selection took place, respecting the following conditions:

- Exclusive to the compound: The study must have the compound as the only studied chemical (an activity value is presented).
- Related to the input keywords: The study title must have at least one of the keywords introduced by the user.
- Conclusive: The result of the bioassay must be either “Active” or “Inactive”, any other results like “Unspecified” or “Inconclusive” were excluded.
- Target protein: There must be an ID of a protein target.

After performing this selection, our dataset was reduced to 10.567 unique compounds, targeting 367 unique proteins. However, several bioassays can involve the same protein-compound pair, and therefore were subsequently removed. As the activity values can vary, a pair was only considered as active if more than 50% of the studies indicate so, the same applies to the inactive, but if it is exactly 50% the pair was taken as inconclusive and removed. This analysis was performed by replacing the activity values by numbers (1 for active and 0 for inactive). As such, we simultaneously consider the positively reported interactions (active) and their counterpart (inactive). The surge of ML-based approaches further stressed out the need to report both positive and negative results, giving rise to new research terms like Structure Inactive Relationships (SIR), which complements the more standard Structure Activity Relationships (SAR) approaches [47]. After performing this final step of pre-processing, the dataset still tallied a total of 10.556 unique compounds and 367 unique proteins.

**Fig. 2:**
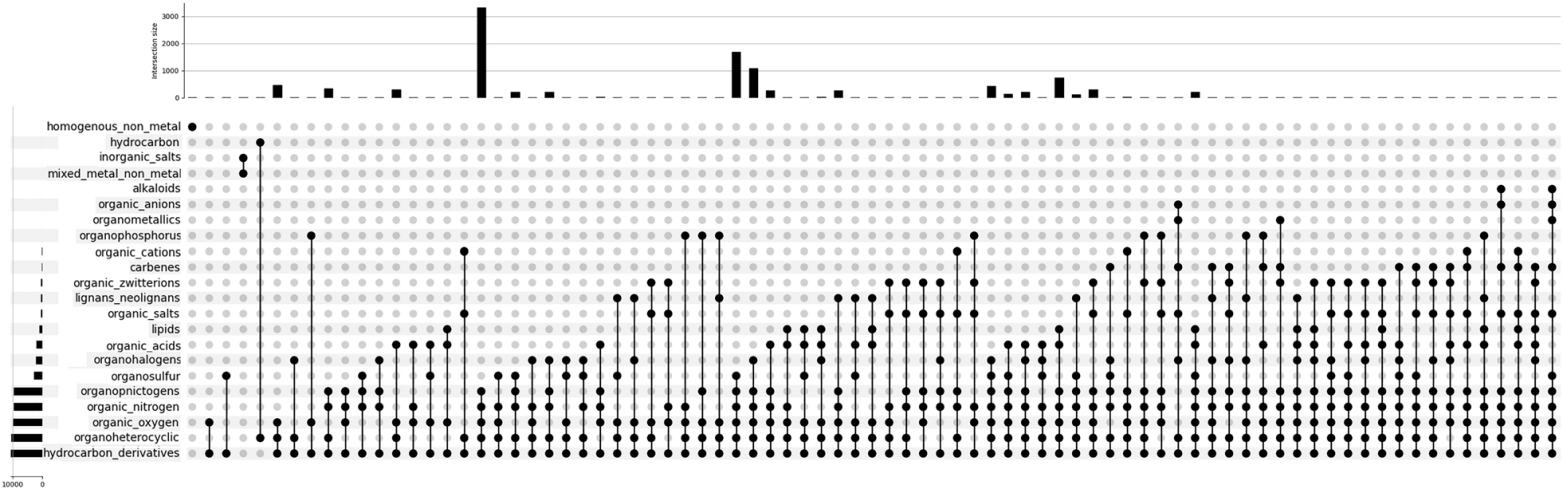
UpSetPlot displaying the bulk analysis of 10.567 unique compounds related to virus research

Finally, it was necessary to retrieve these compounds in a usable format, for which we considered SMILES. A request was conducted PUG-REST [45] returning the isomeric SMILES string of the compound using the CID. Achieving a list of 10.556 SMILES representing unique virus-related compounds, these were tested using our new developed package—DrugTax. Running the DrugTax class on the compounds, their object representation, including superclass categorization and DrugTax features did not exceed 10 s, on a common portable laptop (16 Gb RAM and 11th Gen Intel Core i7-11370H, 3.30 GHz CPU). After retrieving the computed data on table format, we proceeded with the bulk analysis and plotting devices of DrugTax, yielding the UpSetPlot [14] in Fig. 2. As expected, most of the compounds belong to the organic kingdom, although a few exceptions were observed in the form of inorganic salts and/or mixed metal/non-metal inorganic compounds. The most recurring superclass was hydrocarbon derivatives, with few hydrocarbons present (organic molecules containing only carbon and hydrogen). The most populated aggregation of superclasses were organic molecules that fit the superclasses: hydrocarbon derivatives, organoheterocyclic, organic oxygen, organic nitrogen and organopnictogens.

## Applications

DrugTax was developed to simplify molecule characterization. In particular, we deliver a comprehensible molecule categorization as well as clear and humanly interpretable features, which yields a set of simple and fundamental level applications. For example, DrugTax package could be applied to generate similarity searches, chemical space visualization, clustering, taxonomy-property relationships, among others. The results could then be combined with different easy-to-implement visualization tools. For instance, for similarity search, a hierarchical clustering plot could capture the stratified difference between the various molecules. Likewise, for chemical space visualization, by using DrugTax features and projecting the feature vectors into two dimensions with Principal Component Analysis (PCA) or the more recent Uniform Manifold Approximation and Projection (UMAP), users could then produce different scatterplots colored by taxonomic kingdom or superclass.

Due to its easy deployment and installation, DrugTax is a tool whose potential can unfold extensively.

## Conclusions

DrugTax exhibits very fast performance with an easy-to-use interface available on PyPI (https://pypi.org/project/DrugTax/) and GitHub (https://github.com/MoreiraLAB/DrugTax). It extends on the work of Classyfire [13] with novel features oriented towards data science, ML and AI solutions. Its heavily focused on interpretable pharmacological data and features, key for the scientific community, as well as the Pharma sector. DrugTax offers flexible solutions in an intuitive setting that explores the possibilities of SMILES representations for ML and AI solutions on a data-centric setting.

## Supplementary Information

**Additional file 1.** Code Snippets regarding DrugTax.

## Data Availability

DrugTax if of simple installation and usage in any computer that carries Python 3.6.x, with very few dependencies. Most of its extended dependencies emerge when using the bulk analysis and plotting options. Having been deposited in PyPI (https://pypi.org/project/DrugTax/), DrugTax is available through pip installation (C.S.34). Alternatively, DrugTax can be cloned from GitHub (https://github.com/MoreiraLAB/DrugTax). Project name: DrugTax. Project home page: https://pypi.org/project/DrugTax/ Project source code: https://github.com/MoreiraLAB/DrugTax Operating system(s): Platform independent. Programming language: Python. Other requirements: Python 3.6.x or higher. License: GNU GPL.
