# Supplementary material for: DrugTax: package for drug taxonomy identification and explainable feature extraction
Source: J Cheminform. 2022 Oct 27;14:73. doi: 10.1186/s13321-022-00649-w (PMC9609197; doi:10.1186/s13321-022-00649-w)
Supplement: Supplementary file 1 — Additional file 1. Code Snippets regarding DrugTax. [file 13321_2022_649_MOESM1_ESM.docx]

**DrugTax: package for drug taxonomy identification and explainable feature extraction**

A. J. Preto^1,2^, Paulo C. Correia ^3^, and Irina S. Moreira^3,4*^

^1^University of Coimbra, Center for Neuroscience and Cell Biology, 3004-504 Coimbra, Portugal

^2^PhD Programme in Experimental Biology and Biomedicine, Institute for Interdisciplinary Research (IIIUC), University of Coimbra, Casa Costa Alemão, [3030-789](tel:3030-789) Coimbra, Portugal

^3^University of Coimbra, Department of Life Sciences, Calçada Martim de Freitas, 3000-456 Coimbra, Portugal

^4^CNC - Center for Neuroscience and Cell Biology, CIBB - Center for Innovative Biomedicine and Biotechnology, 3004-504 Coimbra, Portugal

* To whom correspondence should be addressed.

Email: [irina.moreira@cnc.uc.pt](mailto:irina.moreira@cnc.uc.pt)

| **Code Snippet 1:** Halogens, metals and group-15/nitrogen atoms lists. |
| --- |

| **Code Snippet 2:** Reading and processing the input SMILE, to yield a version of it with the ordered sequence of atoms. |
| --- |

| **Code Snippet 3:** Replace numbers with the “&” character on an input SMILE, allowing the identification of the atom rings. |
| --- |

| **Code Snippet 4:** Open the file with the input characters, iterate over the possible characters and count their occurrence in an input SMILE. |
| --- |

| **Code Snippet 5:** Outputs a dictionary for later superclass conversion onto features. |
| --- |

| **Code Snippet 6:** Initialize a DrugTax object for an input SMILE. Allows input string or file with single SMILE stored. Computes the atomic sequence, carboxyl identification, ring identification, generates a string SMILE without side group, identifies kingdom and subsequently retrieves superclasses. |
| --- |

| **Code Snippet 7:** Identify whether a compound belongs to the organic or the inorganic kingdoms. |
| --- |

| **Code Snippet 8:** Function to attribute organoheterocyclic superclass. It is also able to identify if compounds are benzenoids. |
| --- |

| **Code Snippet 9:** Function to attribute organosulphur superclass. |
| --- |

| **Code Snippet 10:** Function to attribute the lipid superclass. |
| --- |

| **Code Snippet 11:** Function to attribute the allene superclass. |
| --- |

| **Code Snippet 12:** Function to attribute the allene superclass. |
| --- |

| **Code Snippet 13:** Function to attribute the organic acids superclass. |
| --- |

| **Code Snippet 14:** Function to attribute the alkaloids superclass. |
| --- |

| **Code Snippet 15:** Function to attribute the organic salts superclass. |
| --- |

| **Code Snippet 16:** Function to attribute the organohalogens and organometallics superclasses. Please refer to C.S.1 for the respective lists of atoms. |
| --- |

| **Code Snippet 17:** Function to attribute the organic nitrogen, organic oxygen, and organophosphorus compound superclasses. |
| --- |

| **Code Snippet 18:** Function to identify nucleosides and nucleotides. |
| --- |

| **Code Snippet 19:** Function to identify lignans and neolignans. |
| --- |

| **Code Snippet 20:** Function to identify organic polymers. |
| --- |

| **Code Snippet 21:** Function to identify hydrocarbons and hydrocarbon derivatives. |
| --- |

| **Code Snippet 22:** Function to identify organic anions, cations, and zwitterions. |
| --- |

| **Code Snippet 23:** Function to identify carbenes. |
| --- |

| **Code Snippet 24:** Code to identify organic 1,3-dipolar compounds. |
| --- |

| **Code Snippet 25:** Code to identify organopnictogen compounds. |
| --- |

| **Code Snippet 26:** Code to identify acetylides. |
| --- |

| **Code Snippet 27:** Code to identify homogenous metal compounds. |
| --- |

| **Code Snippet 28:** Code to identify homogenous non-metal compounds. |
| --- |

| **Code Snippet 29:** Code to identify mixed metal/non-metal compounds. |
| --- |

| **Code Snippet 30:** Code to identify inorganic salts. |
| --- |

| **Code Snippet 31:** Download list of SMILEs from ligand name using pubchempy. |
| --- |

| **Code Snippet 32:** Processes a list of input SMILEs, outputs a table with the superclasses for each input SMILE as well as a summary table. |
| --- |

| **Code Snippet 33:** Draw an UpSetPlot using the summary information of a list of drugs. |
| --- |

| **Code Snippet 34:** how to install DrugTax and its dependencies. |
| --- |
